# Computerized adaptive testing of symptom severity: a registry-based study of 924 patients with trapeziometacarpal arthritis

**DOI:** 10.1177/17531934221087572

**Published:** 2022-03-22

**Authors:** Rakhshan Kamran, Jeremy N. Rodrigues, Thomas D. Dobbs, Justin C. R. Wormald, Ryan W. Trickett, Conrad J. Harrison

**Affiliations:** 1 Nuffield Department of Orthopaedics, Rheumatology and Musculoskeletal Sciences, University of Oxford, Oxford, UK; 2 Clinical Trials Unit, Warwick Medical School, Warwick, UK; 3 Welsh Centre for Burns and Plastic Surgery and Swansea University Medical School, Swansea University, Swansea, UK; 4 Cardiff and Vale University Health Board, Cardiff, UK

**Keywords:** Osteoarthritis, patient-reported outcome measures, PROM, computerized adaptive testing, computerized adaptive testing, CAT

## Abstract

We aimed to develop a computerized adaptive testing (CAT) version of the 11 item Patient Evaluation Measure (PEM), using an item response theory model. This model transformed the ordinal scores into ratio-interval scores. We obtained PEM responses from 924 patients with trapeziometacarpal osteoarthritis to build a CAT model and tested its performance on a simulated cohort of 1000 PEM response sets. The CAT achieved high precision (median standard error or measurement 0.26) and reduced the number of questions needed for accurate scoring from 11 to median two. The CAT scores and item-response-theory-based 15-item PEM scores were similar, and a Bland–Altman analysis demonstrated a mean score difference of 0.2 between the CAT and the full-length PEM scores on a scale from 0 to 100. We conclude that the CAT substantially reduced the burden of the PEM while also harnessing the validity of item response theory scoring.

## Introduction

The severity of trapeziometacarpal (TMC) osteoarthritis symptoms can be assessed with patient-reported outcome measures (PROMs), which are self-reported, standardized and validated questionnaires that quantitively measure the elements of health that matter most to patients from their own perspective (Food and Drug Administration, 2009; Weldring and Smith, 2013). PROMs can be used to measure otherwise unobservable or latent traits (e.g. ‘hand function’ or ‘disease severity’) with observable data (e.g. responses to items in a PROM). Infrequent administration poses a marked limitation to the use of PROMs in both research and clinical practice and results in failure to capture important temporal fluctuations in symptom severity. This is particularly important in TMC osteoarthritis, as symptoms vary considerably with activity, time of day, injury and even weather (Dixon et al., 2019; Downing and Davis, 2001; Timmermans et al., 2014). These fluctuations could be captured through more frequent sampling, but the burden of administering lengthy questionnaires at multiple time points may be prohibitive. Response burden can be reduced through computerized adaptive testing (CAT). CAT uses computer algorithms to make PROMs shorter and more patient-centred by selecting the most appropriate items (questions) based on the previous responses (Harrison et al., 2019; Kane et al., 2020). CAT can produce scores that are very similar to the full-length questionnaire. Building a CAT requires two steps: using a dataset of responses to fit an item response theory (IRT) model correctly and then using another dataset to test how well the CAT performs with new users. IRT converts ordinal PROM scores into ratio-interval scores and makes it possible to use parametric statistical methods. Furthermore, it assesses the structural validity of the scoring systems. The Patient Evaluation Measure (PEM) is a hand-specific PROM that can be used across hand surgery (Dias et al., 2001; Macey et al., 1995; Wormald et al., 2019). Part 2 of the questionnaire (as used here) contains 11 items with seven response options on an ordinal scale, with higher scores indicating worse symptoms. While not specific to TMC arthritis, PEM is used for TMC osteoarthritis in national systems like the United Kingdom (UK) Hand Registry and is responsive when used in TMC osteoarthritis (Lane et al., 2020). The aim of this study was to develop and evaluate a CAT version of the PEM for persons with TMC osteoarthritis using an IRT model.

## Methods

### Data collection

Data from 1641 patients with TMC osteoarthritis, who had completed PEM at baseline and at 3, 6 and 12 months post-treatment were obtained from the British Society for Surgery of the Hand (BSSH) UK Hand Registry. This dataset contains 6079 records. These data were originally collected for quality assurance, but secondary uses for research can be approved by the committee managing the registry without requiring further ethical approval for secondary research use. This was confirmed with Oxford University’s Clinical Trials and Research Governance team. Permission to work with the PEM was obtained from the authors who published both existing versions of the PROM (Dias et al., 2001; Macey et al., 1995).

Incomplete response sets (*n* = 3645) were removed. Most of the incomplete response sets were missing responses for item 4 of the PEM (*n* = 3499). Item 4 concerns the duration of pain and was an additional item that was added to the PEM after it was originally developed (Dias et al., 2001). Therefore item 4 was not captured in the UK Hand Registry until 2017, accounting for its absence early on. In total, 2434 response sets from 924 patients were used for the analyses.

## CAT

A CAT algorithm was developed in the R statistical computing environment and evaluated in a Monte Carlo simulation (Harrison et al., 2021). This approach uses the original data to build the model, and then uses computer-generated data to test its performance on a new cohort. To construct the new cohort, we simulated 1000 complete response sets to the full-length PEM questionnaire. For each simulated respondent, the CAT analysed individual responses one at a time, as if it were administering the questions in a real-world scenario. Based on the settings that we used for the CAT, the first question posed was question 8: ‘For everyday activities my, hand is now: no problem > useless’. After each response, the CAT predicted the respondent’s total score, and selected the next most informative item to administer. This continued for each respondent with increasing precision (decreasing standard error of measurement (SEm) around the predicted score) until a score precision threshold of SEm <0.3 was met. At that point, the CAT stopped administering items and the experiment moved on to the next respondent. This precision threshold is comparable with the measurement precision obtained in the Patient-Reported Outcome Measurement Information System (PROMIS) instruments and approximately equates to a marginal reliability of 90% (Gibbons et al., 2011; Reeve et al., 2007).

## IRT

We assessed the structural validity of the PEM and its appropriateness for CAT by evaluating the fit of the UK Hand Registry data to an IRT model, the graded response model. We used comparative fit index; Tucker–Lewis index; root mean square error of approximation; and standardized root mean squared residual statistics to assess fit. The first two of these analyse discrepancies between the data and the hypothesized model, and between the hypothesized model and a null model. High values (close to 1, away from 0) indicate good fit. The latter two analyse the amount of misfit: how far the hypothesized model is from being perfect. Consequently, low values (close to 0) indicate good fit. We considered the following thresholds to indicate good model fit: comparative fit index ≥0.95, Tucker–Lewis index ≥0.95, root mean square error of approximation <0.06, standardized root means squared residual <0.08 (Schreiber et al., 2006). We also used supplementary methods, presented in Appendix S1.

### Measuring CAT performance

For each respondent, three scores were available: the traditional ordinal PEM score (from adding up the raw responses to all of the PEM items), the IRT-based PEM full-length score (from appropriately weighting these responses using the IRT model already described) and the CAT score (which estimated the IRT score, but typically from using fewer items). In addition to the number of items used by the CAT for each of the 1000 simulated respondents, we used the following techniques to determine how closely the CAT-based hand function score reproduced the full-length IRT-based PEM score: (1) Pearson’s correlation coefficient, which indicates the relationship between the CAT and full-length IRT-based scores; (2) mean absolute error between the CAT and full-length IRT-based score; (3) root mean squared error, which is similar to mean absolute error but penalizes individual large (and possibly clinically relevant) errors to a greater extent; and (4) the Bland–Altman method, which calculates the mean difference between the CAT scores versus the full-length IRT-based scores with 95% confidence intervals (Bland and Altman, 1986).

## Results

Demographic and clinical characteristics of the 924 participants are reported in Table 1.

**Table 1.** Characteristics of 924 patients undergoing 959 operations for TMC-osteoarthritis.

Characteristics	Value
Age at operation^a^	64 (12)
Sex	
Male	201 (22)
Female	706 (76)
Missing information	17 (2)
Operation	
Simple trapeziectomy	460 (50)
Trapeziectomy with soft tissue reconstruction or interposition, or with prosthetic spacer	428 (46)
Any other revision procedure for TMC-osteoarthritis	10 (1)
Total joint- or hemi-arthroplasty	48 (5)
TMC-joint denervation	2 (<1)
TMC-joint arthrodesis	8 (<1)
TMC-joint prosthetic ligament reconstruction	2(<1)
TMC-joint stabilization (e.g. Eaton–Littler)	1 (<1)

a Data are presented as years (median (IQR)). All other data are numbers (%).

TMC: trapeziometacarpal.

### Structural validity

Fit statistics, assessed on the simulated cohort, generally demonstrated adequate fit for the CAT (comparative fit index 1.00, Tucker-Lewis index 1.00, root mean square error of approximation 0.11, standardized root means squared residual 0.03). Some of our supplementary results suggest that item 10 (concerning the appearance of the hand) does not combine well with the other PEM items under an IRT model (supplementary Appendix S1). This was not severe enough, however, to justify removing the item from the model.

### CAT performance

**Figure 1.**
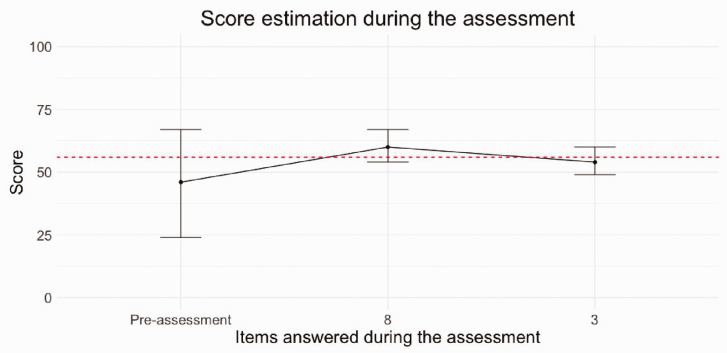
Example of change in score estimation and error for an individual completing the CAT. The score is on the *y*-axis. The person’s true score is 57/100 (red). The current estimate of the person’s score is the black line. As they complete the CAT by first responding to question 8 and thereafter to question 3, the score estimate from the CAT improves, with error (SEm) shown as error bars.

**Figure 2.**
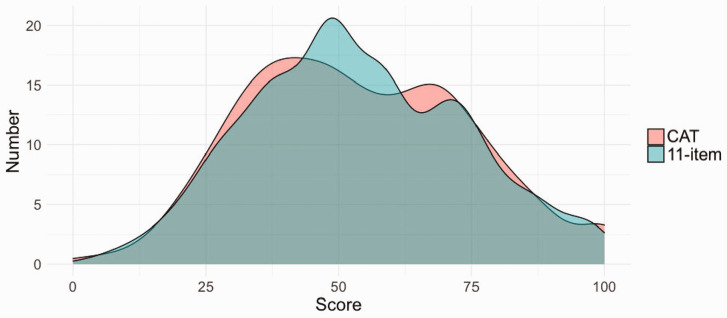
Comparison of CAT, which typically used between 1 and 4 items per individual, to IRT-based PEM scores using all 11 items.

The CAT reduced the number of questions of the full-length PEM from 11 to one to four questions (median two questions) and achieved a high level of precision with a median SEm of 0.26. Figure 1 provides a representation of the CAT’s performance for a person who needed two CAT items to reach score estimate. Before starting the CAT, the estimate of the score is the population average (theta of zero). The first completed item is item number 8 (about everyday activities) where this person selected the impairment as 5 (out of a possible 7), leading to an update in score estimate and reduction in error. The CAT selected item 3 (pain intensity most of the time) as the next most useful item for this person based on the previous response, and the selected response to item 3 was 3. The CAT stopped as error was below the threshold of SEm 0.3. When the theta logit score is converted to a 0–100 score for ease of interpretation, this corresponds to a score of 54/100 from the 2-items used by the CAT. The person’s full-length IRT-based score from all 11 items was 57/100. The distribution of full-length IRT-based PEM scores and CAT scores across the experiment is shown in Figure 2.

**Figure 3.**
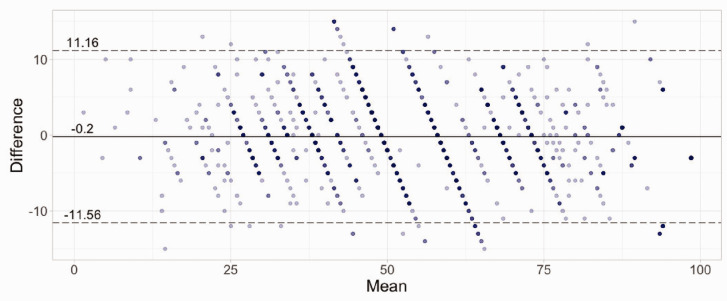
Bland–Altman plot comparing CAT performance with full-length PEM. The mean of the CAT and full-length scores for each individual is on the *x*-axis. The difference between the individuals’ scores is on the *y*-axis. The solid horizontal line shows the mean difference for all scores, and the dashed horizontal lines show the 95% limits of agreement. Increasing intensity in colour of the dots represents multiple data points of the same value overlapping.

CAT scores and full-length IRT-based PEM scores were similar, with a Pearson’s correlation coefficient of 0.96, mean absolute error of 5% and root mean square error of 6% (Figure 2). Bland–Altman analysis (Figure 3) demonstrated a mean score difference of 0.2 between the CAT and the full-length IRT-based PEM scores on a scale from 0 to 100. For 95% of cases, a simulated respondent’s CAT score was between +12 and –11 of the full-length PEM score on the 0–100 scale.

## Discussion

We have developed a CAT version of the PEM that can provide precise outcome measurement (SEm <0.3) in patients with TMC osteoarthritis from one to four (mean two) questions, compared with using all 11 items when deploying the PEM conventionally. This level of precision is comparable with the PROMIS measures deployed through the Epic Electronic Health Records platform (Lapin et al., 2019). Our battery of statistical comparisons has shown that this model is appropriate for calculating PEM scores at both a population level (indicated by the mean error between the CAT and full-length IRT-based score being 0.2) and individual level (as the mean absolute error is 5%).

Our CAT is not only shorter and more individualized than the traditional PEM, the IRT scoring approach means it may have improved structural validity. The traditional PEM is scored on an ordinal scale (i.e. the difference between a score of 19 and 20 is not necessarily the same as the difference between a score of 20 and 21). This is not the case for the CAT, which uses IRT to map scores onto a truly ratio-interval scale through probabilistic modelling. This would mean that, unlike traditional ordinal scoring, they are potentially suitable for parametric statistical analysis, including the presentation of individual scores with a 95% CI.

In our supplementary material, we have provided all the data necessary to operationalize this algorithm as a smartphone application. Hypothetically, if this CAT were deployed through a smartphone, it could facilitate frequent PEM sampling that would provide far richer data than are currently available for clinical practice and research. If this proved to be the case, then one use of such an application could be remote patient monitoring, which may be particularly beneficial in the peri- and post-COVID era (Moynihan et al., 2021; Peek et al., 2020). There is a perception that some older people may not use smartphones and so may not access CATs. They may still need to use full-length paper questionnaires. However, recent data suggest that this may become uncommon. In 2019, 80% of those aged 55–74 in the UK owned a smartphone, and this is a sharply rising trend (Deloitte, 2019).

An assumption of measuring latent traits (e.g. hand function) is that all the items in a questionnaire measure the same underlying construct. This assumption, known as unidimensionality, is tested as part of an IRT analysis. Our analysis showed that PEM item 10 may be different and unrelated to the trait measured by the questionnaire (Supplementary Appendix S1). PEM item 10 asks about hand appearance. At face value, this is quite different from the other items, which ask about sensation and motor function. While appearance may be an important construct to ask, this study suggests that it may be better to assess this separately from the other PEM items. Such potential violations of the unidimensionality assumption are a greater problem for traditional scoring than for CAT. CAT algorithms can often account for poorly discriminant items, and in this study, item 10 was only administered to 11 out of 1000 simulated respondents. In each of these cases, it was presented as the last (and least important) of four questions.

This study has limitations. CAT simulation studies assume that the order in which items are administered does not influence a person’s responses. When this assumption has been investigated in other fields, the order of the items has been shown to have a negligible impact on item responses in IRT-validated scales (Li et al., 2012). While we have used a large dataset of British patients with TMC osteoarthritis, we have not validated this software in patients from other countries or with other conditions.

Future work will focus on determining the acceptability of this technology for frequent, remote symptom monitoring in TMC osteoarthritis as well as application of the PEM CAT for other hand conditions.

## Supplemental Material

sj-pdf-1-jhs-10.1177_17531934221087572 - Supplemental material for Computerized adaptive testing of symptom severity: a registry-based study of 924 patients with trapeziometacarpal arthritis Click here for additional data file. Supplemental material, sj-pdf-1-jhs-10.1177_17531934221087572 for Computerized adaptive testing of symptom severity: a registry-based study of 924 patients with trapeziometacarpal arthritis by Rakhshan Kamran, Jeremy N. Rodrigues, Thomas D. Dobbs, Justin C. R. Wormald, Ryan W. Trickett and Conrad J. Harrison in Journal of Hand Surgery (European Volume)

## References

[bibr2-17531934221087572] Bland JM Altman DG . Statistical methods for assessing agreement between two methods of clinical measurement. Lancet. 1986, 1: 307–10. 2868172

[bibr6-17531934221087572] Deloitte. Mobile Consumer Survey, 2019. Available at: https://www2.deloitte.com/uk/en/pages/technology-media-and-telecommunications/articles/mobile-consumer.html (20 January 2022).

[bibr8-17531934221087572] Dias JJ Bhowal B Wildin CJ Thompson JR . Assessing the outcome of disorders of the hand. J Bone Joint Surg Br. 2001, 83: 235–40. 11284572 10.1302/0301-620x.83b2.10838

[bibr9-17531934221087572] Dixon WG Beukenhorst AL Yimer BB , et al. How the weather affects the pain of citizen scientists using a smartphone app. NPJ Digit Med. 2019, 2: 105. 31667359 10.1038/s41746-019-0180-3 PMC6811599

[bibr10-17531934221087572] Downing ND Davis TRC . Osteoarthritis of the base of the thumb. Curr Orthop. 2001, 15: 305–13.

[bibr11-17531934221087572] Food and Drug Administration. Guidance for industry patient-reported outcome measures: use in medical product development to support labeling claims, 2009. https://www.fda.gov/downloads/drugs/guidances/ucm193282.pdf (accessed 13 September 2021).

[bibr13-17531934221087572] Gibbons LE Feldman BJ Crane HM . Migrating from a legacy fixed-format measure to CAT administration: calibrating the PHQ-9 to the PROMIS depression measures. Qual Life Res. 2011, 20: 1349–57. 21409516 10.1007/s11136-011-9882-y PMC3175024

[bibr15-17531934221087572] Harrison CJ Geerards D Ottenhof MJ , et al. Computerised adaptive testing accurately predicts CLEFT-Q scores by selecting fewer, more patient-focused questions. J Plast Reconstr Aesthet Surg. 2019, 72: 1819–24. 31358447 10.1016/j.bjps.2019.05.039

[bibr16-17531934221087572] Harrison CJ Rodrigues JN Furniss D , et al. Optimising the computerised adaptive test to reliably reduce the burden of administering the CLEFT-Q: a Monte Carlo simulation study. J Plast Reconstr Aesthet Surg. 2021, 74: 1355–401. 10.1016/j.bjps.2020.12.029 33376081

[bibr18-17531934221087572] Kane LT Namdari S Plummer OR , et al. Use of computerized adaptive testing to develop more concise patient-reported outcome measures. JB JS Open Access. 2020, 5: e0052. 32309761 10.2106/JBJS.OA.19.00052 PMC7147635

[bibr19-17531934221087572] Lane JCE Rodrigues JN Furniss D Burn E Poulter R Gardiner MD . Basal thumb osteoarthritis surgery improves health state utility irrespective of technique: a study of UK Hand Registry data. J Hand Surg Eur. 2020, 45: 436–42. 10.1177/1753193420909753 PMC7232779 32162998

[bibr20-17531934221087572] Lapin B Thompson NR Schuster A Katzan IL . Clinical utility of patient-reported outcome measurement information system domain scales. Circ Cardiovasc. 2019, 12: e004753. 10.1161/CIRCOUTCOMES.118.004753 30587028

[bibr21-17531934221087572] Li F Cohen A Shen L Shen L . Investigating the effect of item position in computer-based tests. J Educ Meas. 2012, 49: 362–79.

[bibr22-17531934221087572] Macey AC Burke FD Abbott K , et al. Outcomes of hand surgery. J Hand Surg Br. 1995, 20: 841–55. 8770753 10.1016/s0266-7681(95)80059-x

[bibr23-17531934221087572] Moynihan R Sanders S Michaleff ZA , et al. Impact of COVID-19 pandemic on utilisation of healthcare services: a systematic review. BMJ Open. 2021, 11: e045343. 10.1136/bmjopen-2020-045343 PMC7969768 33727273

[bibr24-17531934221087572] Peek N Sujan M Scott P . Digital health and care in pandemic times: impact of COVID-19. BMJ Health Care Inform. 2020, 27: e100166. 10.1136/bmjhci-2020-100166 PMC7307522 32565418

[bibr25-17531934221087572] Reeve BB Hays RD Bjorner JB , et al. Psychometric evaluation and calibration of health-related quality of life item banks: plans for the Patient-Reported Outcomes Measurement Information System (PROMIS). Med Care. 2007, 45: S22–31. 17443115 10.1097/01.mlr.0000250483.85507.04

[bibr26-17531934221087572] Schreiber JB Nora A Stage FK Barlow EA King J . Reporting structural equation modeling and confirmatory factor analysis results: a review. J Educ Res. 2006, 99: 323–38.

[bibr28-17531934221087572] Timmermans EJ van der Pas S Schaap LA . Self-perceived weather sensitivity and joint pain in older people with osteoarthritis in six European countries: results from the European Project on Osteoarthritis (EPOSA). BMC Musculoskelet Disord. 2014, 15: 66. 24597710 10.1186/1471-2474-15-66 PMC3996041

[bibr29-17531934221087572] Weldring T Smith SMS . Patient-reported outcomes (PROs) and patient-reported outcome measures (PROMs). Health Serv Insights. 2013, 6: 61–8. 25114561 10.4137/HSI.S11093 PMC4089835

[bibr30-17531934221087572] Wormald JCR Geoghegan L Sierakowski K , et al. Site-specific patient-reported outcome measures for hand conditions: systematic review of development and psychometric properties. Plast Reconstr Surg Glob Open. 2019, 7: e2256. 31333975 10.1097/GOX.0000000000002256 PMC6571349

