# Supplementary material for: Computerized adaptive testing of symptom severity: a registry-based study of 924 patients with trapeziometacarpal arthritis
Source: J Hand Surg Eur Vol. 2022 Mar 22;47(9):893–8. doi: 10.1177/17531934221087572 (PMC9535964; doi:10.1177/17531934221087572)
Supplement: sj-pdf-1-jhs-10.1177_17531934221087572 - Supplemental material for Computerized adaptive testing of symptom severity: a registry-based study of 924 patients with trapeziometacarpal arthritis [file sj-pdf-1-jhs-10.1177_17531934221087572.pdf]

Computerised adaptive testing with the Patient Evaluation Measure: a registry based study of 924 patients with trapeziometacarpal arthritis Supplementary Document.

Appendix S1

SUPPLEMENTARY METHODS

Unidimensionality

Prior to the item response theory analysis, we evaluated unidimensionality through exploratory factor analysis (EFA) and confirmatory factor analysis (CFA).

Exploratory Factor Analysis

We tested the following assumptions for EFA: multivariate normality using Mardia's test; sampling adequacy using the Kaiser-Meyer-Olkin (KMO) test; and correlation adequacy using Bartlett's test.

To determine the number of factors, we produced a scree plot and conducted a parallel analysis, and Kaiser criterion analysis with a threshold of 1.0 eigenvalues. Very simple structure (VSS) analysis and Velicer's minimum average partial (MAP) were also determined.

Confirmatory Factor Analysis

A confirmatory model was fit using the *lavaan* package (v0.6-9) in R and evaluated with the following fit statistics: Comparative Fit Index (CFI); Tucker-Lewis index (TLI); Root Mean Square Error of Approximation (RMSEA); and Standardized Root Mean Squared Residual (SRMR). We used the following thresholds to indicate

good model fit: CFI ≥ 0.95, TLI ≥ 0.95, RMSEA < 0.06, SRMR < 0.08. (Schreiber et al., 2006; Hu and Bentler, 1999).

Monotonicity

Mokken analysis, including the automatic item selection procedure, was used to assess monotonicity, with a Loevinger's H Statistic value of >0.3 assumed to indicate scalability (van der Ark, 2007).

Item-level and Scale-level Fit Statistics

A graded response model (GRM) was fitted using the mirt package in R. Item-level fit statistics (infit and outfit) were computed in addition to scale level fit statistics (Linacre, 2002).

Local dependence

We checked for local dependence using a Yen's Q3 residual covariance matrix. We considered a threshold of >0.2 to demonstrate local dependency (Christensen et al., 2017).

Measurement Invariance

We performed differential item functioning (DIF) with nested logistic regression models and likelihood ratio tests to check the assumption of measurement invariance (Choi et al., 2011).

SUPPLEMENTARY RESULTS

Unidimensionality

Exploratory Factor Analysis:

Item responses violated the assumption of multivariate normality. Correlation and sampling were adequate (Table S1).

Table S1. EFA Assumptions and Testing

Assumption for EFA	Test Used	Test Result
Multivariate normality	Mardia's test	Non-normal
Sampling adequacy	KMO test	0.96
Correlation adequacy	Bartlett's test for sphericity	31320.81 (55), $p < 0.01$

The Scree Plot (Figure S1), Kaiser criterion analysis, VSS analysis, and Velicer's MAP (Table S2) suggest a 1-factor model.

Figure S1. Scree plot demonstrating a 1-factor model

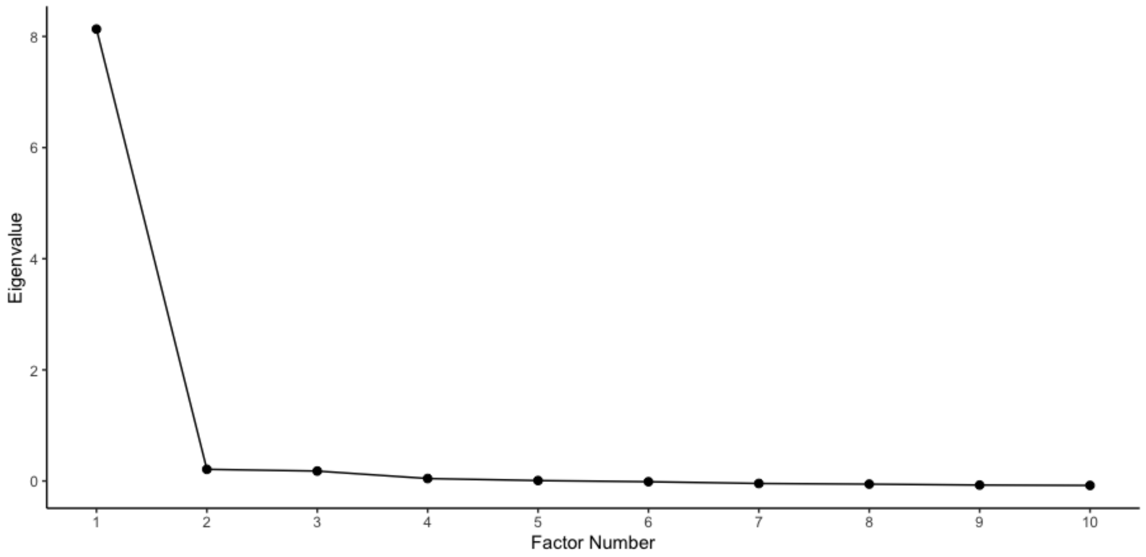

Table S2. IRT Assumptions and Model Fit Results

Analyses	Statistic	Result
IRT assumptions and model fit		
Exploratory Factor Analysis	Scree Plot	Suggests 1 Factor
	Parallel Analysis	Suggests 2 Factor
	Kaiser Criterion Analysis (factors with >1.0 eigenvalues)	Suggests 1 Factor
	MAP	Suggests 1 Factor
	CFI	0.998

Confirmatory Factor Analysis	TLI	0.998
	RMSEA	0.109
	SRMR	0.033
Local Dependency	Yen’s Q3 Residual Covariance >0.2	4 item pairs locally dependent
Monotonicity for Scale	Loevinger’s H Statistic	0.766

Confirmatory Factor Analysis

Results from CFA are reported in Table S2.

Monotonicity

Results from Mokken analysis are displayed in Table S2.

Item-level and Scale-level Fit Statistics

Item- and Scale-level fit statistics are displayed in Table S3 and Table S4.

Table S3. GRM parameters

ID	a	b1	b2	b3	b4	b5	b6

PEM1	3.203	-1.103	-0.410	0.101	0.547	1.107	1.613
PEM2	3.497	-1.212	-0.459	0.042	0.456	0.983	1.656
PEM3	4.848	-0.894	-0.200	0.213	0.615	1.167	1.745
PEM4	3.670	-1.287	-0.332	0.112	0.492	0.904	1.258
PEM5	3.888	-1.385	-0.725	-0.261	0.086	0.482	0.878
PEM6	4.029	-0.856	-0.312	0.091	0.491	0.919	1.388
PEM7	3.456	-1.558	-0.764	-0.268	0.177	0.601	1.033
PEM8	6.290	-1.031	-0.434	0.028	0.492	1.049	1.650
PEM9	4.170	-1.050	-0.476	-0.027	0.465	0.988	1.555
PEM 10	1.708	0.046	0.594	0.978	1.365	1.948	2.568
PEM 11	3.152	-0.495	0.002	0.364	0.746	1.225	1.748

Table S4. Infit and Outfit Statistics for PEM Items

ID	Outfit	Infit
PEM1	1.036	1.083
PEM2	0.937	0.976
PEM3	0.875	0.899
PEM4	0.937	1.002
PEM5	0.939	0.959
PEM6	1.046	1.034
PEM7	0.981	0.982
PEM8	0.789	0.863
PEM9	1.101	1.123
PEM 10	0.780	0.947
PEM 11	0.884	1.011

Local Dependence

We found 4 pairs of locally dependent items, 3 of which had mild values of Yen's Q3 between 0.23-0.24 with 1 pair having a value of 0.37 (item 10, which relates to hand appearance, and item 11, which relates to how patients feel about their hand) (Table S5).

Table S5. Locally Dependent Item Pairs

Item Pairs	Yen's Q3 Value
Item 2 and Item 3	0.24
Item 3 and Item 4	0.23
Item 5 and Item 7	0.23
Item 11 and Item 10	0.37

Measurement Invariance

Gender was known for 2390 participants. Likelihood ratio tests between logistic regression models suggested that items 5, 9, and 10 demonstrated uniform DIF by gender ($p < 0.001$).

Supplementary References

Choi SW, Gibbons LE, Crane PK. lordif: An R Package for Detecting Differential Item Functioning Using Iterative Hybrid Ordinal Logistic Regression/Item Response Theory and Monte Carlo Simulations. J Stat Softw. 2011, 39: 1–30.

Christensen KB, Makransky G, Horton M. Critical Values for Yen's Q3: Identification of Local Dependence in the Rasch Model Using Residual Correlations. Appl Psychol Meas. 2017, 41: 178–94.

Hu L, Bentler PM. Cutoff criteria for fit indexes in covariance structure analysis: Conventional criteria versus new alternatives. Structural Equation Modeling: A Multidisciplinary Journal. 1999, 6: 1–55.

Linacre, J. M. What do Infit and Outfit, mean-square and standardized mean? Rasch Measurement Transactions. 2002, 16, 878.

Schreiber JB, Nora A, Stage FK, Barlow EA, King J. Reporting Structural Equation Modeling and Confirmatory Factor Analysis Results: A Review. The Journal of Educational Research. 2006, 99: 323–38.

van der Ark, LA. Mokken Scale Analysis in R. Journal of Statistical Software. 2007, 20(11), 1–19. https://doi.org/10.18637/jss.v020.i11
